# The Influence of Color on the Consumer’s Experience of Beer

**DOI:** 10.3389/fpsyg.2017.02205

**Published:** 2017-12-19

**Authors:** Felipe Reinoso Carvalho, Pieter Moors, Johan Wagemans, Charles Spence

**Affiliations:** ^1^Department of Marketing, School of Management, Universidad de Los Andes, Bogotá, Colombia; ^2^Brain and Cognition, Faculty of Psychology and Educational Sciences, KU Leuven, Leuven, Belgium; ^3^Crossmodal Research Laboratory, Department of Experimental Psychology, University of Oxford, Oxford, United Kingdom

**Keywords:** beer, flavor, color, crossmodal correspondences, sensory marketing, multi-sensorial

## Abstract

Visual appearance (e.g., color) cues set expectations regarding the likely taste and flavor properties of food and drink. These expectations may, in turn, anchor the subsequent tasting experience. In the present study, we examined the influence of the color of a beer on the consumer’s experience. Dark and pale beers were evaluated both before and after tasting. Importantly, these beers were indistinguishable in terms of their taste/flavor when tasted without any visual cues. The results indicate that the differing visual appearance of the beers led to clear differences in expected taste/flavor. However, after tasting, no differences in flavor ratings were observed, indicating that the expectations based on visual cues did not influence the actual tasting experience. The participants also expected the dark beer to be more expensive than the pale one. These outcomes suggest that changes in the visual appearance of a beer lead to significant changes in the way in which consumers expect the beer to taste. At the same time, however, our findings also suggest the need for more evidence to be collected in order to determine the boundary conditions on when such crossmodal expectations may vs. may not affect the tasting experience.

**Highlights:** The expected flavor of a beer is affected by its visual appearance. No differences in flavor ratings were observed on tasting. Consumers expect dark beers to be more expensive than pale/amber beers.

## Introduction

Over the last 80 years or so, several hundred studies have assessed the influences that visual cues (such as color) have on the experience of different food and drink items (e.g., Moir, 1936; Zellner et al., 1991; Parr et al., 2003; Zellner and Durlach, 2003; Zampini et al., 2007; Spence, 2015b; see Spence and Piqueras-Fiszman, 2014, for a review). For instance, the color of a food/drink (or the color of its packaging; Barnett and Spence, 2016; Lick et al., 2017) can influence which products consumers notice and, consequently, which they choose to buy (i.e., color influences shopping behavior), as well as influence their tasting experience.

The consumer’s interaction with food or drink usually starts prior to the tasting experience itself, creating a rich context for the development of sensory/hedonic expectations (see Piqueras-Fiszman and Spence, 2015, for a review). Actually, the interaction between colors and taste/flavors, in the case of taste and flavor perception, should not be understood only at the time that tasting occurs. The role of higher-level cognitive factors (e.g., consumer expectations) should also be considered (Shankar M. et al., 2010). It has been argued that under the appropriate conditions, both sensory congruency and sensory incongruity between what is expected and what is experienced while tasting can bring positive or negative reactions to the overall tasting experience (Stevenson, 2009; Spence et al., 2010; Piqueras-Fiszman and Spence, 2012; Spence, 2016; Velasco et al., 2016a; Yanagisawa, 2016). Visual cues (particularly color) set sensory and hedonic expectations regarding the likely taste and flavor properties of food and drink items. The suggestion is that these expectations may, in turn, anchor the subsequent tasting experience (e.g., Garber et al., 2000; Demattè et al., 2006; see Spence et al., 2010; Piqueras-Fiszman and Spence, 2015, for reviews).

Color-taste/flavor associations have been studied in a number of different ways. For instance, by comparing the presence versus absence of color, or by changing the characteristics of the color that is present in a food or drink (e.g., its intensity, hue, etc.). Other studies, meanwhile, have studied how the expectations triggered by food coloring influence the judgment of a food or a drink’s flavor (see Spence et al., 2010, for a review). Researchers have demonstrated that the four or five well-known basic tastes (bitter, sweet, sour, salty, and umami) are individually associated with particular colors as well (e.g., Koch and Koch, 2003; Velasco et al., 2016a; see Spence et al., 2015, for a review), and that such crossmodal associations can be exploited in the context of consumer behavior (Garber et al., 2001, 2003a,b). For example, by adding yellow coloring to a sweet solution, it is possible to significantly decrease people’s sensitivity to sweetness (Maga, 1974). There is also a growing awareness that cultural differences can influence the way in which people establish color–flavor associations (Shankar M.U. et al., 2010; Velasco et al., 2014; Wan et al., 2014a,b; Jacquot et al., 2016; Wan et al., 2016).

Guided by the aforementioned literature review, we wondered whether such ideas could be studied using a particular type of food, or drink, available in a wide range of colors that happens to be consumed frequently in everyday life. Therefore, we decided to work with beer. There is great diversity associated with the flavor of beer, and some of these flavor attributes are more commonly associated with a blond, pale, or perhaps a dark beer. However, such flavor diversity is not necessarily constrained within a certain color category. Nevertheless, we asked ourselves how people would judge a beer’s flavor when comparing, let us say, two beers of very different colors, but very similar flavor. Could particular sensations triggered by the differences in color be transferred to the tasting experience, in a way that would lead to significantly different flavor judgements (see Cheskin, 1972, on the notion of sensation transference)?

Previous research has already approached the role of visual cues in a beer tasting experience as when, for instance, comparing the same beers under blind versus sighted tasting conditions. When given access to both visual aspects of beer (appearance and brand identity), consumers tend to report different preferences (Allison and Uhl, 1964; Guinard et al., 2000). Hedonic ratings also seem to change from blind to similar informed tasting conditions (Guinard et al., 2001; Lee et al., 2006), with significant variations across different age ranges (Guinard et al., 2001). Meanwhile, elsewhere, it has been shown that differently colored beer labels can also exert a significant influence over consumers’ hedonic and flavor ratings, as well as on their purchase intent, even when drinking the beer from the glass, i.e., away from the packaging (Barnett and Spence, 2016). A recent report also measured the impact of affect, and the senses, in the experience of drinking beer in real context situations (see Gómez-Corona et al., 2017). These results revealed no significant differences in expected liking and purchase intent between the eight beers that were evaluated. However, semantic differences were reported between those phrases that resembled the more cognitive aspects of a beer experience (that were more frequently associated with craft beer types), versus phrases that indexed the more sensory and affective aspects of beer (and which were more frequently associated with industrial beers).

In the experiment reported here, participants tasted two beers sighted. Both beers were produced in order to be indistinguishable in terms of their flavor (when tasted without visual cues), but at the same time to have a very different visual appearance. One beer, which fell within a pale/amber color range, was intended to represent a kind of ale/lager beer type (pale beer). The other, darker, was intended to visually simulate a Belgian style double (abbey Trappist), or perhaps a porter type color range (i.e., a very dark type of beer). Note that the baseline beer used to create both drinks was produced following the standards for blond-ale beer, which, speaking flavor-wise, represents a filtered, light (in terms of alcohol and body), and hoppy beer.

In this study, we wanted to know whether by creating a dark version of a light beer, it would be possible to significantly influence the tasting experience, when comparing the expected and actual tasting sensory/hedonic evaluations of participants. Our main objective was to try and gain some understanding of the perceptual implications of experiencing a dark beer, which would be most likely judged as incongruent in terms of flavor, and especially when compared to a pale one. On the one hand, we were interested in the potential perceptual influence that the visual appearance of the beer might have on people’s flavor judgments (e.g., Shankar M.U. et al., 2010). On the other hand, we were particularly interested in understanding how the contrasting formula of the dark beer would affect the consumer’s tasting experience, and the expected price of such a product (i.e., by triggering confirmation and/or disconfirmation of expectation responses; see Oliver, 1977; see also Hovland et al., 1957; Piqueras-Fiszman and Spence, 2015, for an overview on assimilation and contrast effects). This experiential exercise was set as a comparison between such dark beer and its pale counterpart.

When thinking about the potential applicability of these ideas, we detected the dramatic increase of the microbrewery movement^1^, particularly in markets where the production of craft beers is, by no means, historically common - e.g., located outside Europe. The exponential rise of the demand for craft beers is providing great access – and triggering great interest – in the consumption of more unconventional types of beer, such as dark ones (Carroll and Swaminathan, 2000; Murray and O’Neill, 2012).

## Materials and Methods

### Participants

Between the 16th and 18th of December, 2016, visitors at the Musical Instruments Museum Brussels (MIM), in Belgium, were invited to take part in a short experiment. They were informed that they would be given complimentary beer to taste while answering a short survey. A total of 136 participants agreed to take part (45% females, 55% males, mean – *M* – age of 32.3 years, standard deviation – *SD* – of 12.4, with around 60% of participants being between 20 and 30 years old, and a total of 80% of participants being less than 40 years old). All of the participants were at least 16 years of age (the minimum legal age to drink beer in Belgium), and gave their informed consent prior to taking part in the study. None of the participants reported having a cold or any other impairment of their senses of smell, taste, or hearing at the time of the study. In general, the participants were mostly European residents, with the majority from Belgium (28.7%), France (19.0%), and the United Kingdom (18.4%).

### Stimuli

The beers that were used in this experiment were produced under the strict supervision of the Laboratory of Enzyme, Fermentation and Brewing Technology, at KU Leuven, Belgium. Here, an in-house-produced blond-ale beer was used as baseline (with 7 EBC; EBC^2^ is the color based standard reference method of color grade in beers; a higher EBC means a darker colored beer, and vice-versa^3^). The color grade of this baseline beer was artifically altered in two ways, in order to obtain two beers with different colors, but similar flavor. In summary, one beer was fermented and filtered (baseline). Afterward, two batches were colored separately and then individually carbonated/bottled.

**FIGURE 1:**
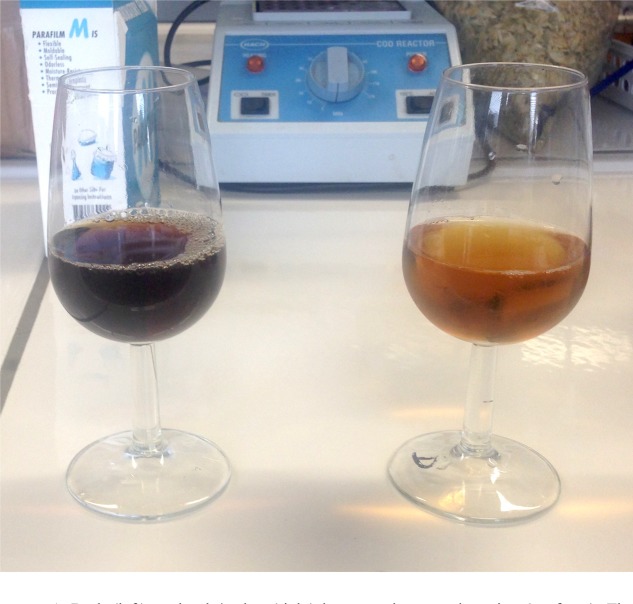
Dark (Left), and pale/amber (Right) beers – prior to carbonation (no foam). The beer was light in body and alcohol, but with strong hoppiness. Three types of hops were used to make the beer, resulting in a fine bitterness and light kettle hop aroma, combined with spicy and floral notes from traditional hops, used in late hopping. While being light in alcohol and body, this beer potentially offered full aroma and taste/flavor impressions. The raw materials used for coloring both beers were the same (but, of course, were added in different quantities). Therefore, significant changes were expected mostly on the intensity of the resultant colors (e.g., Chroma). Note that, besides the artificial coloring process, both beers were fermented and brewed following the same standards, which most likely resulted in similar haziness/cloudiness and head characteristics (foam).

The color agents used for this process were provided by PureMalt^4^ (color agents labeled as RB7, RB1500). These coloring agents were chosen to have a minimum impact on the resulting flavor. The two resultant beers were referred to as pale (17.5 EBC), and dark (50 EBC). The dark beer resulted from mixing 2.9 g of RB1500 plus 2.4 g of RB7 per liter of the original 7 EBC. The pale beer formula was the result of mixing 45% of 20 EBC (the latter was obtained by adding 1.4 g of RB7 per liter on the original 7 EBC), 45% of original 7 EBC, plus 10% of the dark – 50 EBC. The resultant formulas outputted two beers with similar-low body, smell, hopiness, and astringency. The final alcohol content of the two beers was 5.5 (% v/v), with a bitterness of around 22 IBU^5^, and a carbonation level of 5.6 g/l. The suggested temperature for pouring these beers was between 5 and 10° Celsius (this temperature range was maintained during the entire experimental procedure). All of the beers used in the present study were consumed during a period of no more than 6 months after bottling. **Figure 1** shows the two resultant beer colors.

Following industry standards, a triangle test was implemented for defining participants’ ability to differentiate between the resultant flavors of the two beers. The triangle test is a discriminative method commonly used in the sensory evaluation of foods and drinks (see British Standard ISO, 2004, for details). In this test, the participants had to choose which beer was different from three choices, without knowing anything about which beer they were tasting each time (since they could not actually see the differences in what they were drinking^6^). Twenty five participants took part in this test (the British Standard ISO, 2004 standard recommends a minimum of 18 assessors for triangle testing differences). Here, three darkened-and-numbered-cups were filled with two different beers (two cups filled with the same beer, and the third cup with a different beer; in our case, a distribution between our pale and dark beers). This pouring process was counter-balanced across participants. The resultant ratings were compared by means of a Pearson Chi-square distribution. The results confirmed that the participants were not able to demarcate the differences in flavor between the pale and dark beers (*X*^2^_10,05_ = 2.163, *p* = 0.141). These results therefore suggest that naïve drinkers would not be able to differentiate the flavor of the beers that were produced here (when tasted without visual cues), leaving their differences in color as the most important factor that could lead to changes in their consuming behavior.

### Experimental Design and Procedure

The Social and Societal Ethics Committee at KU Leuven (SMEC) approved this protocol (registered as G-2015 09 339).

#### Experimental Design

The objective of this particular study was to compare the expectations and the actual taste/flavor judgment of each participant (using a within-participants experimental design, with two conditions). The experiment took place on the 9th floor of the MIM. During the three experimental days, it was possible to have a well-controlled and stable environment, although more naturalistic as compared to a laboratory environment. The experiments were performed using computers placed on tables. The participants sat together, although fairly well separated, to prevent them from interacting with one another. Each participant joined the experiment for approximately 10 min.

#### Experimental Procedure

Each participant was seated in front of a computer screen with a computer mouse, and a keyboard to complete the survey. Each participant had two transparent-plastic cups filled with the dark and pale beers, respectively, with each cup containing no more than 5 cl. Before and after drinking the beers, the participants were advised to drink tap water, for palate cleansing (a cup filled with tap water was available for each participant).

The survey consisted of an electronic form containing three main stages. In the first step, the participants were instructed to read and accept the conditions of the informed consent before entering their demographic details. In a second step, the participants responded to a pre-questionnaire in which they rated their expectations concerning the taste (bitterness, sweetness), flavor (body, alcohol strength), and expected liking of the pale and dark beers. In the third step of the procedure, the participants tasted each beer, while again answering the same questions as in the second step (the beers were experienced separately, meaning that the participants answered the corresponding batch of questions after tasting each of the two beers). Steps two and three consisted of seven-point scales presented in a randomized order (with the number 1 of the scale representing ‘not at all,’ the number 4 representing ‘balanced’/‘moderated’/‘neutral’ - depending on the question; and ‘7’ representing ‘very much’; for example, 1 for ‘not at all bitter,’ 4 for ‘moderate,’ and 7 for ‘very bitter’ ratings). Finally, the participants indicated which of the two beers they preferred, and which beer they thought was the most expensive (here, order-randomized multiple-choice questions were used). Note that only the color ratings were based on a bipolar scale (with 1 being ‘very dark,’ 4 being ‘moderate,’ and 7 ‘very pale’).

The order of presentation of the beers was counterbalanced across participants. Hence, the participants were advised to follow the survey instructions carefully, in order to drink the appropriate beer at the appropriate time. Note that there were always at least two supervisors present during the entire experimental process for extra guidance, coordination, and support, in addition to the self-guiding written experimental-instructions. Upon finishing the study, the participants were instructed to leave the room without discussing any details with the next group of participants.

#### Analysis

As the data for each rating scale were based on a 2 × 2 (pre-taste vs. post-taste by dark vs. pale) within-subjects experimental design, we subjected the data to a 2 × 2 repeated measures ANOVA for each scale, separately. As the analysis of many scales might not guarantee proper Type I error control for α = 0.05, we applied a conservative Bonferroni correction for multiple comparisons. In addition to considering the six scales, we also consider the three statistical tests conducted in each ANOVA as potentially inflating Type I error (see Cramer et al., 2016). Therefore, we set α = 0.05/18 = 0.00277 for our main analyses reported below. As a measure of effect size, we report generalized η^2^ as proposed by Olejnik and Algina (2003). All analyses were conducted in R, using the RStudio IDE, and mainly relying on the *tidyverse* and *afex* packages.

## Results

### Expected versus After-tasting Ratings

**FIGURE 2:**
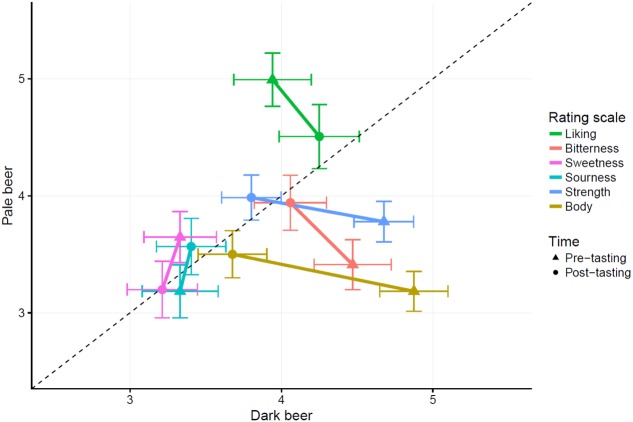
Scatterplot depicting average ratings for each combination of beer type, time and rating scale. Error bars indicate means ±2 SEM. The dotted line is the identity line. Points closer to the dotted line indicate average ratings for both beer types are similar. Points below the dotted line indicate higher ratings for the dark beer, while points above the line indicate higher ratings for the pale beer.

**Figure 2** summarizes the mean ratings of the participants prior to (triangles), and after (circles) tasting the dark and pale beers.

For liking, we observed a main effect of beer type [*F*(1,135) = 25.42, *p* < 0.0001, $\eta_{G}^{2}$ = 0.05], no main effect of time [*F*(1,135) = 0.89, *p* = 0.35, $\eta_{G}^{2}$ = 0.0009], and an interaction effect between beer type and time [*F*(1,135) = 15.73, *p* = 0.0001, $\eta_{G}^{2}$ = 0.02]. As can be seen from **Figure 2**, the pale beer was liked more, on average. However, this main effect was qualified by an interaction which indicated that the liking ratings actually converged. Indeed, when considering the simple effects, participants expected to like the pale beer more than the dark beer [*t*(253.66) = 6.415, *p* < 0.0001] prior to tasting, yet after tasting there was no difference [*t*(253.66) = 1.57, *p* = 0.12].

A similar pattern of results emerged for bitterness ratings. Again, a main effect of beer type was observed [*F*(1,135) = 20.63, *p* < 0.0001, $\eta_{G}^{2}$ = 0.04]. There was no main effect of time [*F*(1,135) = 0.34, *p* = 0.56, $\eta_{G}^{2}$ = 0.0005], and there was an interaction effect between beer type and time [*F*(1,135) = 16.4, *p* < 0.0001, $\eta_{G}^{2}$ = 0.03]. On average, the dark beer was expected to be more bitter than the pale one, yet this main effect was again qualified by an interaction indicating that the corresponding ratings converged. Prior to tasting, the dark beer was expected to taste more bitter than the pale beer [*t*(266.88) = 6.085, *p* < 0.0001]. Yet, after tasting, the aforementioned difference disappeared [*t*(266.88) = 0.676, *p* = 0.5).

For sweetness ratings, no main effect of beer type was observed [*F*(1,135) = 1.44, *p* = 0.23, $\eta_{G}^{2}$ = 0.003]. A main effect of time was observed, but did not survive the correction for multiple comparisons [*F*(1,135) = 8.56, *p* = 0.004, $\eta_{G}^{2}$ = 0.01]. No interaction between beer type and time emerged [*F*(1,135) = 2.21, *p* = 0.14, $\eta_{G}^{2}$ = 0.004].

Similarly for sourness ratings, no differences emerged in the analysis either for the main effects of beer type [*F*(1,135) = 0.005, *p* = 0.94, $\eta_{G}^{2}$ < 0.0001] or time [*F*(1,135) = 4.51, *p* = 0.04, $\eta_{G}^{2}$ = 0.007] nor for the interaction [*F*(1,135) = 2.13, *p* = 0.15, $\eta_{G}^{2}$ = 0.003].

For the ratings of strength, we observed a main effect of beer type [*F*(1,135) = 14.16, *p* = 0.0003, $\eta_{G}^{2}$ = 0.03], a main effect of time [*F*(1,135) = 18.18, *p* < 0.0001, $\eta_{G}^{2}$ = 0.02], and an interaction between beer type and time [*F*(1,135) = 36.85, *p* < 0.0001, $\eta_{G}^{2}$ = 0.06]. The main effects indicated that the dark beer was rated as stronger on average compared to the pale beer, and that the expected strength was higher prior to tasting compared to after tasting. This interpretation should again be qualified by the results of the interaction, which again converges to the results of the previous scales. Prior to tasting, the dark beer was expected to taste stronger compared to the pale one [*t*(268.95) = 6.898, *p* < 0.0001], yet this difference (prior to tasting) was no longer observed when comparing the after-tasting ratings of both beers [*t*(268.95) = 1.414, *p* = 0.16].

The same pattern emerged for the ratings of body. Main effects of beer type [*F*(1,135) = 73.14, *p* < 0.0001, $\eta_{G}^{2}$ = 0.13] and time [*F*(1,135) = 20.65, *p* < 0.0001, $\eta_{G}^{2}$ = 0.03] as well as an interaction between beer type and time were observed [*F*(1,135) = 64.81, *p* < 0.0001, $\eta_{G}^{2}$ = 0.09]. The dark beer was rated to have more body, on average, as compared to the pale beer, and ratings of body were higher prior to tasting compared to after tasting. The interaction depicts a similar pattern, where the dark beer was expected to have more body compared to the pale one [*t*(264.22) = 11.734, *p* < 0.0001], yet this difference again disappeared after tasting [*t*(264.22) = 1.224, *p* = 0.22].

In summary, for each of the six scales, we observed a clear and consistent pattern. Wherever prior expectations about the flavor of the beer were apparent based on the visual appearance, such expectations-based differences did not affect the corresponding ratings after having tasted the beer, since the after-tasting ratings of both beer types tended to converge.

Finally, 43.5% of the participants reported that they expected the dark beer to be more expensive than the pale beer. On the other hand, 28.0% reported the opposite, and 28.5% reported that both beers should have the same price. A Pearson’s chi-square goodness-of-fit test for distribution conducted between ‘dark,’ ‘pale,’ and ‘same price’ choices – with confidence of 95% – shows a weak, but significant difference between these three choices [χ^2^(2) = 6.191, *p* = 0.045].

In the following section, we report an exploratory analysis that was inspired by our main analysis. That is, we wanted to verify whether the convergence observed in the after-tasting ratings would generalize across countries (here, we mainly consider Belgian versus British versus ‘other’ participants for reasons outlined below)^7^. Because this analysis was conducted *post hoc*, we merely report it to inspire new research, and do not commit ourselves to strong conclusions based on these results.

### Cross-Country Comparison

Given the fact that dark Belgian beers are usually very different, in terms of their flavor profile, than dark British beers (for instance, some dark Belgian beers may be twice as strong as their British counterparts), and considering that Belgian and British participants were amongst the most well-represented nationalities within our population^8^ – and both have a historical tradition of producing and consuming dark beers – the data was subdivided into three sub-groups (Belgians, with *N* = 39, British, with *N* = 25, and others, with *N* = 71).

**FIGURE 3:**
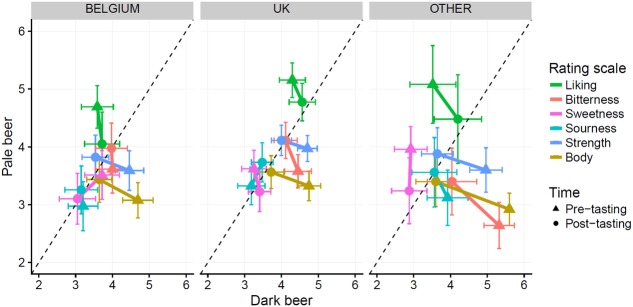
Scatterplot depicting average ratings for each combination of beer type, time and rating scale, split up by country. Error bars indicate means ±2 SEM. The dotted line is the identity line. Points closer to the dotted line indicate average ratings for both beer types are similar. Points below the dotted line indicate higher ratings for the dark beer, while points above the line indicate higher ratings for the pale beer.

To analyze if there were significant differences in the ratings across these sub-groups, a repeated measures ANOVA was calculated, with country as the between-participant variable (Belgium, United Kingdom, and others), type of beer/time as within-independent variables, and the rating scales as dependent variables (see **Figure 3**). For succinctness, we only report the effects that reached statistical significance (refer to the Supplementary Material^9^ for the complete results of the analysis). For liking, a main effect of country group was observed [*F*(2,132) = 6.42, *p* = 0.002, $\eta_{G}^{2}$ = 0.04]. Here, participants from Belgium gave lower ratings, on average, as compared to those from the UK and from other countries. For bitterness ratings, an interaction between country group and color was observed [*F*(2,132) = 8.88, *p* = 0.0002, $\eta_{G}^{2}$ = 0.04]. This interaction effect largely emerged from the participants from ‘other’ countries expecting the dark beer to be more bitter prior to tasting, allowing them to regress more to the similar average ratings that were obtained for dark and pale beers after tasting. For all other scales, no pronounced effects of country group were observed.

The price judgment across the different subgroups of countries was also visualized. **Table 1** shows this comparison. Here, most Belgians and ‘others’ tend to expect the dark beer to be more expensive than the pale one (values in bold). On the other hand, the corresponding results for the British participants do not show such a trend. Nevertheless, these percentage differences did not reach statistical significance [χ^2^(4) = 2.33, *p* = 0.68].

**Table 1:** Participants’ price judgment for the beers, across the different three sub-groups of countries (Belgium, United Kingdom, and others).

Which beer is more expensive?		
Belgium	Dark	51%
	Pale	23%
	Same price	26%
UK	Dark	36%
	Pale	36%
	Same price	28%
Others	Dark	42%
	Pale	28%
	Same price	30%

### Correlations

**FIGURE 4:**
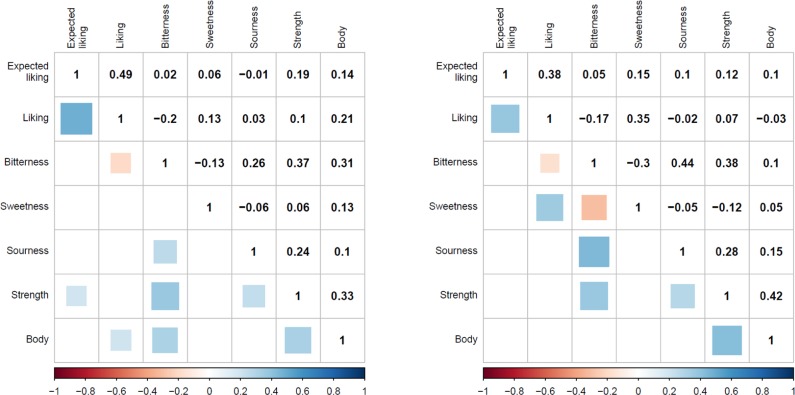
Correlations between ratings after tasting for pale beers (Left) and dark beers (Right).

**Figure 4** shows the correlations across the ratings for the pale, and dark beer, respectively. The upper panel depicts the numerical correlations values, while the lower panel denotes the correlations that remain statistically significant after Holm correction. Among the significant results we can appreciate that, for both beer types, expected liking and after-tasting liking ratings are positively correlated. For both beers, bitterness is negatively associated with liking. For dark beers sweetness is positively related to liking, but negatively to bitterness. For both beers, sourness and bitterness, strength and bitterness, strength and sourness, and body and strength are all positively related. Last, for pale beers, body was positively associated with liking and bitterness.

## Discussion and Future Work

The participants in the present study evaluated the flavor of two beers having distinctly different appearances (one very dark and another pale/amber), but indistinguishable in terms of their flavor (when tasted without visual cues). The sensory/hedonic evaluation of the two beers occurred both before and after tasting. Here, we hypothesized that by creating different color versions of the same drink (in this case, two different color versions of a light beer) it would be possible to significantly influence the tasting experience (Moir, 1936; Demattè et al., 2006; Spence et al., 2010). These results suggest that there can be differences between what the participants expect from both beers, and their judgment after tasting them, yet more evidence is needed in order to show that these expectations could, in fact, influence the overall tasting experience.

In terms of expectations, the participants expected to like the pale beer more than the dark one. The dark beer was expected to be more bitter, to taste stronger, and to have more body than the pale beer (see **Figure 2**). Even though blond/pale beers are most commonly regarded as more filtered, with stronger hoppiness, and more bitter, than dark ones, it has been previously shown that for certain beverages (including beers), darker brown colors are associated with stronger, or more bitter, tastes/flavors (Guinard et al., 1998).

When comparing these expectations with after-tasting ratings across both beer types, no significant differences were observed. The latter result suggests that the differences in color did not affect the way the participants judged the beers after tasting. Such a lack of crossmodal effects suggests that the visual expectations were discounted when it came to the tasting experience. Hence, we could somehow conclude that color differences in beers do not have a significant impact on a beer’s flavor judgement. In other words, people do not seem to rely on beer color categories while denoting a beer’s flavor, at least when the expectations set by eye (regarding a beer’s taste/flavor attributes) turn out to be a long way from the actual tasting experience.

As a matter of fact, for our experiment in particular, the most salient flavor cues of the dark beer were mostly incongruent (bitterness, alcohol strength and body), since the formula of this dark beer was based on the brewing process of a blond-light beer. Here, we hypothesize that the contrast between what was expected and what was experienced could have weakened any possible transference of sensations, from the process of expectations into the tasting experience itself (Cheskin, 1972). The latter would be in line with the theory of assimilation-contrast (Hovland et al., 1957). This theory claims that if the difference between expectation and reality is somehow within a person’s limit of acceptance, it would be possible to, consciously or not, change the perceptual evaluation of a food/drink product in order to bring it in line with the expectations (see Piqueras-Fiszman and Spence, 2015, for a review). However, if such a difference is sufficiently great, a consumer would tend to exaggerate this difference between expectations and reality, shifting the product’s evaluation toward the opposite direction, when compared to what was originally expected (Schifferstein, 2001; Yeomans et al., 2008; Wang et al., 2017).

Interestingly, the color of these beers affected their expected price. Here, the consensus was that the dark beer was expected to be more expensive than the pale one (see Expected versus After-Tasting Ratings) – this was reported by almost half of the sample. The subsequent cross-cultural comparison of Section “Cross-Country Comparison” suggested that the British residents did not show the same price judgment tendencies when compared to the other two groups of participants (Velasco et al., 2014, 2016a,b; Wan et al., 2014a,b; Jacquot et al., 2016). Yet due to the relatively low frequencies, no statistical evidence was obtained for this trend. Nevertheless, we believe that this cross-cultural assessment may inspire future analysis of correlation between the darkness of a beer and its price, across the markets of very different countries and/or regions.

In future research, it might be interesting to investigate whether a congruently darkened beer formula might actually lead to a different tasting experience, when compared to its blond/pale counterpart. For instance, by somehow matching the expectations, a darkened pale-strong beer^10^ may be judged as even stronger, and so on (see Gottfried and Dolan, 2003; White and Prescott, 2007; Reinoso Carvalho et al., 2015, 2016a,b, 2017; see Spence, 2011, for an overview on how crossmodal congruency can lead to perceptual enhancement in different multisensory experiences). Breaking down the color characteristics of a beer in more detail (e.g., by comparing differences in hue spectrum, or differences in haziness/cloudiness) could also help better disentangling the perceptual influence that a beer’s color can have on the tasting experience (cf. Barnett et al., 2017). Given recent work showing that the shape of glassware can influence the tasting experience as well (e.g., Spence et al., 2012; Spence and Piqueras-Fiszman, 2014; Spence, 2015a; see Spence and Van Doorn, 2017, for an overview), it is necessary to bear in mind that the color of a beer is never experienced in isolation, but is often affected by the glassware/material (e.g., plastic, glass) in which it is presented^11^. Concerning the limitations of the experiment reported here, we did not take into account the participants’ initial beer preferences. Such differences may potentially bias the visual scoring and consequent taste ratings. We also chose to compare the expectations and the actual taste/flavor ratings of each of the participants while experiencing both beers (within-participant design). However, in our everyday experience of beer we do not necessarily compare two different beers in detail while choosing between them. We further ask ourselves if an experimental design where each participant would drink the same beer twice, blind versus sighted, would provide similar results (when compared to this method that uses triangle testing instead). Following the latter thought, future experiments may also consider comparing, for instance, a blind-tasting versus informed tasting conditions, in order to compare the effects of colour along with semantic-(in)congruency oriented analyses (e.g., Guinard et al., 2000, 2001).

## Ethics Statement

This study was carried out in accordance with the recommendations of ‘The Social and Societal Ethics Committee at KU Leuven (SMEC),’ with written informed consent from all participants. All participants gave written informed consent in accordance with the Declaration of Helsinki. The protocol was approved by The Social and Societal Ethics Committee at KU Leuven (SMEC) – protocol registered as G-2015 09 339.

## Author Contributions

FR, JW, and CS designed the study. FR collected the data and participated in the design of the experimental stimuli. PM lead the data analysis, with the participation of FR. All authors participated in manuscript preparation and all authors revised the final version of the manuscript.

## Conflict of Interest Statement

The authors declare that the research was conducted in the absence of any commercial or financial relationships that could be construed as a potential conflict of interest.

## Footnotes

1 See https://www.ft.com/content/c9f77348-8ccc-11e6-8cb7-e7ada1d123b1 (retrieved August, 2017).

2 See Beer 10-A Spectrophotometric Color Method, ASBC (American Society of Beer Chemists) methods of analysis, for details on the EBC methodology.

3 See https://tinyurl.com/beer-ebc-scale for an overview of the EBC standardized scale (retrieved April, 2017).

4 See http://www.puremalt.com for details of the company and their products.

5 IBU is the International bitterness units in Wort, obtained by segmented flow analysis. See Method Wort-23, of the ASBC (American Society of Beer Chemists) methods of analysis.

6 During the triangle test, the beers were served in darkened-black cups. The foam of the resultant dark beer was somewhat darker than that of the pale one. Therefore, in order to prevent any color bias, the participants were blindfolded while tasting. The lights in the experimental room were set at the minimum.

7 A similar analysis was carried out across gender. Similarly to the assessment across country groups, only a main effect of gender was observed for liking ratings [*F*(1,133) = 15.6, *p* = 0.0001, $\eta_{G}^{2}$ = 0.05]. Here, men tended to give higher average liking ratings compared to women. In summary, the differences in rating across gender do not seem to affect the overall results, since men and women follow similar judgment trends. Refer to the Supplementary Material for the complete results of the analysis (see footnote 9).

8 In this experiment, the French were also among the highest number of participants. The French, however, are not known for having a specific beer culture as far as dark beers are concerned.

9 Complete data analysis, in .html and .pdf formats, are available through the following link: https://doi.org/10.6084/m9.figshare.5687830.v1 (retrieved on November, 2017).

10 For example, traditional Belgian brewers such as Duvel Moortgat (http://www.duvel.com/fr-be/la-biere/duvel), and Abdij der Trappisten Westmalle (http://www.trappistwestmalle.be/nl/de-tripel-van-westmalle?agecheck=plus18) are well-known for offering strong pale beers (retrieved March, 2017).

11 Several breweries customize their glassware in many different ways, as part of their branding strategies; i.e., http://www.telegraph.co.uk/men/thinking-man/11765323/Does-the-shape-of-your-glass-affect-how-a-beer-tastes.html (Retrieved 19th April, 2017).
